# Dart and the Taung juvenile: making sense of a century-old record of hominin evolution in Africa

**DOI:** 10.1098/rsbl.2024.0185

**Published:** 2024-07-24

**Authors:** John Rowan, Bernard Wood

**Affiliations:** ^1^ Department of Archaeology, University of Cambridge, Cambridge CB2 3DZ, UK; ^2^ CASHP, Department of Anthropology, George Washington University, Washington, DC 20052, USA

**Keywords:** paleoanthropology, human evolution, *Australopithecus*

## Abstract

The announcement in 1925 by Raymond Dart of the discovery of the Taung juvenile’s skull in a quarry in sub-Saharan Africa is deservedly a classic publication in the history of palaeoanthropology. Dart’s paper—which designated Taung as the type specimen of the early hominin species *Australopithecus africanus*—provided the first fossil evidence supporting Charles Darwin’s 1871 prediction that Africa was where the human lineage originated. The Taung juvenile’s combination of ape and human characteristics eventually led to a paradigm shift in our understanding of human evolution. This contribution focuses on the milieu in which Dart’s paper appeared (i.e. what was understood in 1925 about human evolution), the fossil evidence as set out by Dart, his interpretation of how a species represented by a fossilized juvenile’s skull fitted within prevailing narratives about human evolution and the significance of the fossil being found in an environment inferred to be very different from that occupied by living apes. We also briefly review subsequent fossil finds that have corroborated the argument Dart made for having discovered evidence of a hitherto unknown close relative of humans, and summarize our current understanding of the earliest stages of human evolution and its environmental context.

## 1. Context

In 1871, Charles Darwin—influenced by the anatomical similarities between African apes and modern humans demonstrated by Thomas Henry Huxley [1]—predicted that ‘it is somewhat more probable that our early progenitors lived on the African continent than elsewhere’ [2]. In 1925, this prediction must have seemed wide of the mark, for at the close of the nineteenth century Europe was the primary source of fossil evidence for human evolution—the exceptions being the Talgai cranium from Australia and Dubois’ discoveries in Java. Fossil evidence relevant to the later stages of human evolution had been recovered in Africa prior to 1925: in 1914 at Boskop in what is now the Gauteng Province of South Africa, and in 1921 at Broken Hill (now Kabwe) in what was then Northern Rhodesia (now Zambia) [3]. The Boskop 1 calvaria and the Kabwe 1 cranium and limb bones, both close enough to modern humans to be assigned to the genus *Homo* as *Homo capensis* [4] and *Homo rhodesiensis* [3], respectively, were a long way from being the African ‘early progenitor’ predicted by Darwin. As we explain below, in 1925 it must have seemed that Ernst Haeckel—not Charles Darwin—may have been more prescient, for Haeckel believed that the extant apes from Southeast Asia (gibbons and orangutans) are more closely related to modern humans than the extant African apes. If we apply Darwin’s logic, according to Haeckel the fossil evidence of our ‘early progenitors’ would more likely be located in Southeast Asia than in Africa.

Influenced by Ernst Haeckel’s ideas about where our ancestors originated, Eugène Dubois, a medical doctor, had travelled to Southeast Asia, and in April 1890 he was given permission to look for human ancestors on the island of Java. Although Dubois’ team found a fragmentary mandible at Kedoeng Broebos (now Kedung Brubus) in the Kendeng Hills, he redirected his survey effort towards the sediments exposed on the banks of the Solo River in central Java, near the village of Trinil. In September 1891, Dubois’ team recovered an upper third molar, Trinil 1, and in his third quarterly report to the Dutch East Indies Government, Dubois allocated the tooth to *Anthropopithecus*, the genus name then used for extant chimpanzees and for the fossil apes then being recovered by Richard Lydekker near the present-day border area of India and Pakistan. One month later, Dubois’ team recovered a partial cranium, Trinil 2, and the following year they discovered a complete adult left femur, Trinil 3. Dubois initially assigned the Trinil fossils to a novel species, *Anthropopithecus erectus*, but in 1894 he changed the genus to *Pithecanthropus*, a name Haeckel introduced in his 1868 book *Natürliche Schöpfungsgeschichte* (*The History of Creation*) [5,6]. The Trinil 2 calotte, which combined a small cranial capacity with a low braincase and a sharply angulated occipital region, was judged to be more primitive than the only other extinct hominin known at the time, *Homo neanderthalensis*.

When Dart announced the Taung^1^ juvenile—with its small brain and relatively modern human-like teeth and jaws—as a candidate for Darwin’s ‘early progenitor’ he had to push back against two opposing forces. The first was the prevailing wisdom that Dubois had already unearthed evidence of an ‘early progenitor’ in Southeast Asia. The second was the implication of Charles Dawson’s ‘discovery’ of *Eoanthropus dawsoni* in England in a gravel pit near the Sussex village of Piltdown [7]. Dawson claimed his discoveries at Piltdown demonstrated that our ‘early progenitors’ had large brains and ape-like jaws. It was much later that Weiner *et al*. [8] confirmed what some had long suspected: Piltdown was a forgery. The combination of primitive, ape-like jaws and a large brain was the result of someone—almost certainly Dawson—deliberately ‘planting’ parts of a modern human brain case along with the lower jaw of an orang-utan in the gravel pit. De Groote *et al*. [9] provided an updated analysis of the source of Dawson’s Piltdown ‘evidence’.

## 2. Dart’s description and interpretation of the fossil evidence

**Figure 1.**
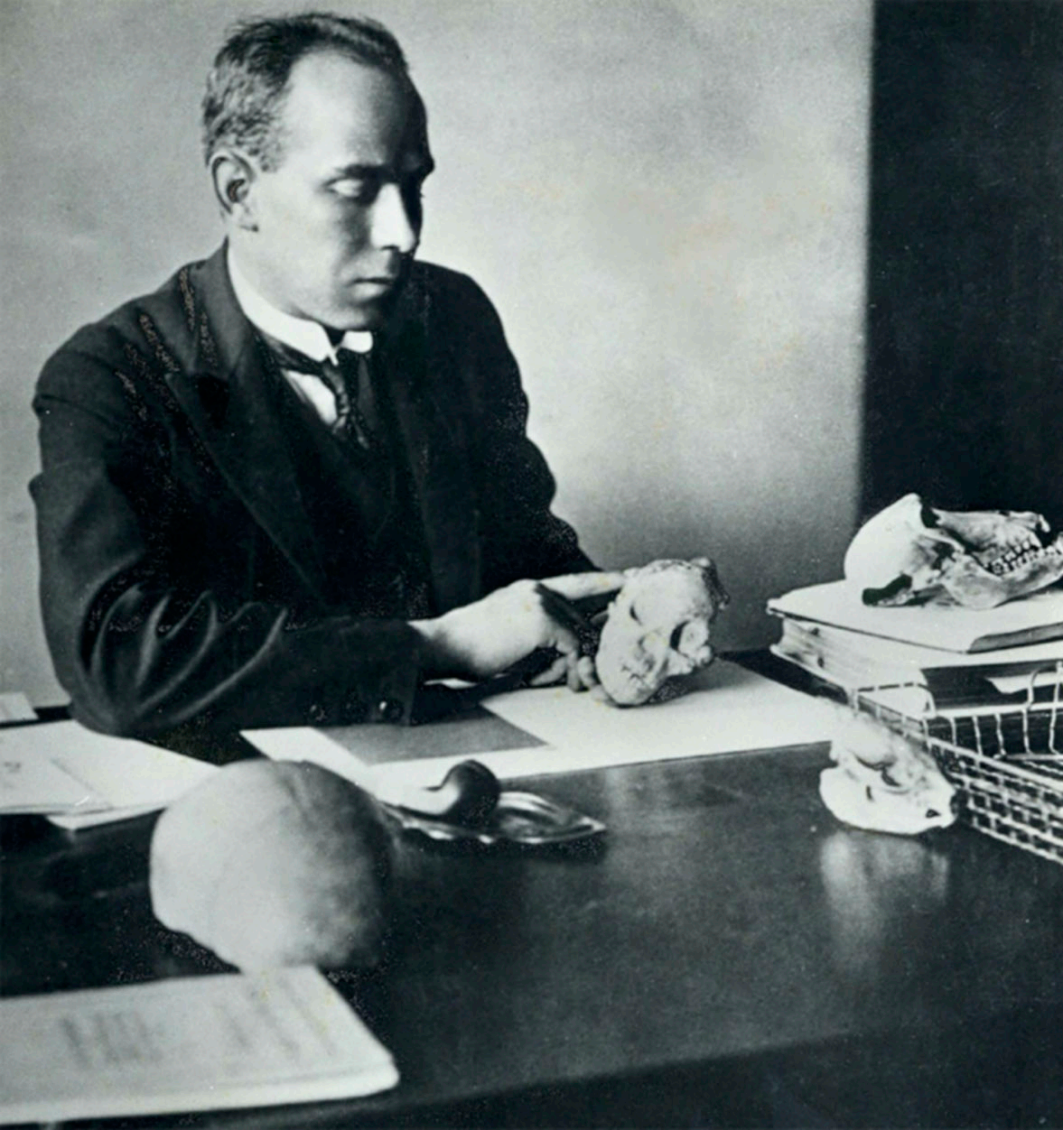
Raymond Dart with the Taung juvenile shortly after the publication of his account in *Nature* [11] in February 1925.

The circumstances of the discovery of Taung 1 and how it was brought to Dart’s attention at the end of November 1924 are set out elsewhere [10]. After removing the breccia rock attached to the fossil, Dart became convinced the juvenile skull belonged to a human-like ape and not the baboons that were common in the cave sediments at Taung (figure 1). Dart summarized the results of his rapid comparative analysis in a manuscript he mailed to the editor of *Nature* on 6 January 1925. The editor’s response was swift and positive, and Dart’s manuscript was published in the form of a ‘letter’ in the 7 February issue of *Nature* [11].

Although Dart was just 29 years old when he was appointed to the Chair of Anatomy at the University of the Witwatersrand in 1922, his apprenticeship with the anatomist Grafton Elliot Smith at University College London, and his visits to many of the top anatomy departments in the USA during his time as a Rockefeller Foundation Fellow, meant he was one of the few people who could appreciate the significance of the cranial, mandibular, dental and endocranial morphology of the Taung juvenile [12]. In his *Nature* letter, Dart explained that although ‘fragments of the distal ends of the forearm bones’ and ‘phalanges were present in the rock mass from which the facial fragment was isolated’, ‘these proved too fragmentary and too friable to develop but portions are still visible in the stone’ [13, p. 319], so the skull and the natural endocranial cast (i.e. brain-cast) were all that remained of the Taung juvenile’s skeleton.

Dart’s letter takes the reader through the cranium’s overall shape, the morphology of the dentition, mandible and dental arcade and the location of the foramen magnum. The natural endocranial cast’s size and surface morphology had mostly faithfully captured the relative size of the major lobes of the brain, as well as the pattern of gyri and sulci. Dart did not finally separate the jaw from the cranium until 1929, so in 1924, all that could be seen of the tooth crowns were the labial surface of the incisors and canines and the buccal surface of the postcanine teeth.

After distinguishing between an ‘ape-like man’ (*sensu Pithecanthropus erectus*) and a ‘man-like ape’, Dart proposed that the Taung juvenile belonged to a new genus and species of human-like ape, *Australopithecus africanus*, which he allocated to a novel family, *Homo-simiadæ.* As for the factors that allowed *Au. africanus* to persist in what he inferred to be an ‘untoward environment’ [11] consisting of ‘open veldt country’ (*ibid.*, p. 199) some ‘two thousand miles…from its nearest anthropoid cousins’ living in ‘luxuriant forests’ (*ibid.*, p. 198), Dart opted for intellect and, to a lesser extent, bipedality. He suggested that the ‘relative scarcity of water, together with a fierce and bitter mammalian competition furnished a laboratory’ that had helped to ‘sharpen the wits’ and promote ‘adroitness of thinking and movement’—elements that allowed the Taung juvenile and its kin to survive and thrive. Dart cited the relatively anterior placement of the foramen magnum as evidence that *Au. africanus* placed ‘greater reliance…upon the feet as organs of progression’, thus freeing the hands from their role as ‘accessory organs of locomotion’ (*ibid.*, p. 197). To Dart, this suggested that the hands of the Taung juvenile had assumed a ‘higher evolutionary role’ as ‘examining organs’ capable of performing ‘elaborate, purposeful, and skilled movements’, as well as being organs of ‘offence and defence’ that took the place of ‘massive canines and hideous features’ (*ibid.*, p. 197) that had been lost.

When it was published in 1925, Dart’s letter in *Nature* fell ‘like a bombshell on anthropological Europe’, as one anatomist put it [12]. Dart’s paper was accompanied by brief commentaries contributed by three distinguished anatomists, Sir Arthur Keith, Sir Grafton Elliot Smith and Wynfrid Duckworth, and an equally distinguished palaeontologist, Sir Arthur Smith Woodward, along with a longer separate commentary by Sir Arthur Keith. Although Wynfrid Duckworth was of the opinion that because the Taung juvenile ‘came immediately under notice of so competent an anatomist as Prof. Dart establishes confidence in the thoroughness of the scrutiny’ [14], the criticisms the commentators expressed focused on the challenges posed by the Taung individual’s juvenile status and the fossil’s ill-defined geological context. One commentator suggested the features Dart had used to show that ‘*Australopithecus* was nearly akin to man’ were owing to ‘the youthfulness of the specimen’, claiming they were ‘essentially identical with the conditions met in the infant gorilla and chimpanzee’ [14]. The commentators also suggested that better geological evidence will ‘help settle its relationships’ and provide ‘geological evidence of age’.

A century later, we recognize Dart’s conclusion that the Taung juvenile represented an ‘extinct race of apes intermediate between living anthropoids and man’ [11, p. 195] as remarkably perceptive, especially considering the difficulties he faced in analysing an immature skull with a comparative sample of extant taxa that was essentially restricted to adult material. Dart’s assessment of the Taung skull depended heavily on his conclusions about the relative size of the face and the dentition. Specifically, Dart noted that the reduced (as compared with living apes) height of the upper and lower canine crowns and the small size of the gap (aka diastema) between the upper lateral incisor and the upper canine pointed to the dentition being ‘humanoid rather than anthropoid’ (*ibid*., p.196). Dart also drew attention to the robust (i.e. relatively wide for its height) mandibular corpus and the un-buttressed (i.e. there are no obvious transverse ridges of bone to support it) and vertical mandibular symphysis as additional evidence of the Taung juvenile’s human affinities. Dart explicitly contrasted the more human-like morphology of the Taung mandibular symphysis with that of Piltdown, noting that in this aspect ‘*Eoanthropus dawsoni* scarcely differs from the anthropoids’ (*ibid*., p. 197). We can now see this as another one of Dart’s shrewd assessments, for Piltdown was not formally exposed as an elaborate hoax until 1953.

## 3. Taung to the present-day—*Australopithecus* and *Australopithecus africanus*

What is the status of Dart’s new genus and species a century after their announcement? Has more fossil evidence strengthened, or weakened, support for Darwin’s prediction that our early progenitors lived on the African continent? Is *Au. africanus* still our ‘earliest progenitor’?

**Figure 2.**
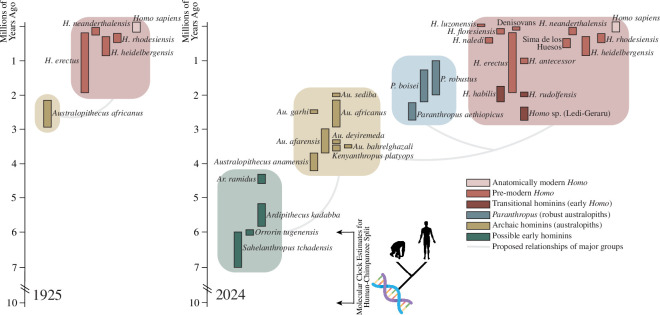
The hominin fossil record, as known in 1925 (left) and 2024 (right). Modified from Wood & Boyle [16].

Since the recognition of *Australopithecus* in 1925, fossil evidence from existing and additional sites in southern Africa and sites in eastern and central Africa has resulted in the recognition of at least two (*Australopithecus afarensis* and *Australopithecus garhi*) and possibly as many as six (*Australopithecus anamensis*, *Australopithecus bahrelghazali*, *Australopithecus deyiremeda*, *Australopithecus* (*Kenyanthropus*) *platyops*, *Australopithecus prometheu*s and *Australopithecus sediba*) additional *Australopithecus* species ([15]; figure 2).

As for *Au. africanus*, although no more hominins have been recovered from the Nolan Limeworks at Buxton (the locality from which the Taung juvenile derives), specimens that most researchers allocate to *Au. africanus* have been recovered at three other cave sites—Makapansgat [17], Sterkfontein [18] and Gladysvale [19]—in southern Africa. All are far from Taung; Makapansgat is located to the northeast of Johannesburg, whereas Sterkfontein and Gladysvale are in the Blauuwbank valley, equidistant from Johannesburg and Pretoria.

Of the three sites mentioned, Sterkfontein is the most productive [20]. Before being recognized as a fossil site, the Sterkfontein cave system had been commercially explored for lime, and most recently for guano. In 1936, Trevor Jones, one of Dart’s students, visited Cooper’s hardware store in Krugersdorp—between Johannesburg and Pretoria—where he noticed some vertebrate fossils that were part of the display advertising guano from Sterkfontein. When Jones visited Sterkfontein, George Barlow—who had worked for a time at the Nolan Limeworks at Taung—was selling fossils from a table, and during a later visit by another of Jones’ teachers, Gerrit Schepers, and a fellow student, William Harding Le Riche, Barlow gave the visitors a fossilized baboon mandible. After these fossils were drawn to Robert Broom’s attention, he also visited Sterkfontein. Broom asked Barlow to tell him if any Taung-like fossils were found, and just a week later in July 1936 Broom was called back by Barlow who showed him TM (for Transvaal Museum) 1511, the first hominin recovered from Sterkfontein. Broom suggested the TM 1511 fragmentary cranium ‘probably agrees fairly closely with the Taungs ape,’ but he thought ‘it advisable to place the new form in a distinct species,’ *Australopithecus transvaalensis* [21]. Following the onset of WWII, work at Sterkfontein was suspended until Broom and his assistant John Robinson resumed excavations in 1947. Since then the search for fossils from Sterkfontein has been more or less continuous, resulting in the recovery of more than 600 hominin specimens.

Compared with Sterkfontein, Makapansgat and Gladysvale have yielded more modest fossil hominin samples. Dart [17], who reported the first hominin specimens from Makapansgat, designated an occipital fragment—MLD (for Makapansgat Limeworks Deposit) 1—the type specimen of a new species, *Australopithecus prometheus.* This species name reflected Dart’s belief that the Makapansgat hominins made and used fire.

Although other genus names were introduced for the southern African fossil hominins, primatologist Sherwood (Sherry) Washburn and palaeontologist Bryan Patterson suggested that the taxonomy of the fossil samples from Taung, Sterkfontein and Makapansgat could be simplified [22] and their proposal to use *Australopithecus* as the only genus name received influential support from both John Robinson [23] and Wilfrid Le Gros Clark [24]. Others carried taxonomic simplification a step further, with *Au. africanus* Dart 1925—the nomen with taxonomic priority—now widely considered to subsume both *Au. transvaalensis* Broom 1936 and *Au. prometheus* Dart 1948 as junior synonyms.

Clarke [25] and colleagues (e.g. [26]) have challenged this long-standing taxonomic consensus by reviving the ‘two morph’ interpretation of *Australopithecus* samples from Sterkfontein and Makapansgat, with some specimens (e.g. Sts 5, 17, 19, 52 and MLD 6, 18, 40) allocated to *Au. africanus* and others (e.g. Sts 7, 36, 71, StW 252, 431, 505, 573 and MLD 1, 2, 9) allocated to *Au. prometheus*. Other researchers familiar with the fossil evidence [27–29] do not support partitioning the *Au. africanus* hypodigm in this—or any other—way. Likewise, although two partial hominin skeletons from Malapa were designated as the type series of a novel species, *Australopithecus sediba* [30], the juvenile status of MH1 (now U.W. 88-1), plus the many cranial similarities with *Au. africanus*, suggest that Malapa, dated to 1.98 Ma [31], may sample a more recent part of the *Au. africanus* lineage [32]. Most of the breccia deposits providing the hypodigm of *Au. africanus*—Taung, Sterkfontein (Members 4 and 2), Makapansgat (Members 3 and 4A) and Gladysvale—broadly span the 3–2 Ma interval [33]. Recent research has suggested that the Member 2, Member 4 and Jacovec Cavern breccias at Sterkfontein may be as old as 4–3.5 Ma [34,35], but this older age is inconsistent with biochronological evidence (e.g. [36,37]).

A century after its discovery, Dart’s *Au. africanus* now stands as the best-known (and possibly the only) *Australopithecus* species from the southern Africa cave deposits. A notable challenge to this consensus came from Tobias [38], who, influenced by the recently revised age estimates for the Sterkfontein and Makapansgat fossil assemblages [39], suggested that the perceived younger age of the Taung deposit implied that the juvenile skull might instead be linked to fossils attributed to *Paranthropus robustus.* Tobias’ claim prompted re-evaluations of the affinities of the Taung juvenile, but its dental [40], basicranial [41], facial [42] and endocranial [43] morphology all link it with the abundant hominin remains from Sterkfontein and Makapansgat.

## 4. Taung and the environments of early human evolution

Dart [11] is usually recognized as the publication that established the ‘savannah hypothesis’, but models in which hallmark human adaptations—such as large brains and bipedalism—evolved in the context of open, non-forested environments had been suggested at least a century beforehand (reviewed in [44]). Darwin [2, pp. 140–141] suggested that humans originated when ‘an ancient member in the great series of Primates’ experienced ‘a change in the conditions of its native country, to live somewhat less on trees and more on the ground’, resulting in modification to ‘its manner of progression’, and Lamarck [45] had expressed similar ideas in his *Philosophie zoologique*. These models of human origins were likely well known to Dart. For example, 2 years before Dart’s report appeared in *Nature*, Keith [46, p. 451] recounted that Lamarck ‘regarded the erect posture [of humans] as a result of the chimpanzee-like ancestor having abandoned an arboreal mode of life for one in the open country’ and he was ‘fully alive to the fact that any anthropoid which had acquired the human mode of progression had gained an enormous advantage; it would no longer be confined to tracts of tropical jungle but would have the whole length and breadth of the earth open to it’.

Given this background, it seems likely that Dart was preconditioned to situate his ‘man-ape’ in an ‘open veldt country’ (*ibid.*, p. 199). A year after announcing the Taung discovery, Dart defended and expounded on his ideas for an ‘entirely terrestrial man-ape phase’ of human evolution that played out in the context of environments that were uninhabitable by the ‘lumbering anthropoids of the tropics’ [13, pp. 315–316]. In fact, after arguing that the available evidence supported the persistence of open, arid landscapes in southern Africa since the Cretaceous and the region’s long-term environmental separation from the equatorial forest belt, Dart went as far as to suggest that—even without fossils—geography alone would have been sufficient to reconstruct this critical phase of human evolution:

If we had possessed no endocranial cast and, consequently, no corroborative evidence concerning the increased intelligence, the better use of the hands and the more erect stature of [*Au. africanus*] from the actual remains themselves, these matters might reasonably have been inferred from a consideration of the geographical site at which the discovery was made [13, p. 318].

The dichotomy of ‘ape’ versus ‘human’ environments became entrenched in palaeoanthropological discourse, such that when faced with the same lines of contextual evidence, individuals came to very different conclusions about the environment of the Taung juvenile according to their views on the fossil’s place in human evolution. For example, Keith’s book *New Discoveries Relating to the Antiquity of Man* (1931 [47]) positioned *Au. africanus* as an ‘extinct cousin of the gorilla and chimpanzee’, and therefore Dart’s characterization of the Taung environment was necessarily flawed. According to Keith, an ape equals a forest and *vice versa*:

If we suppose that the forest belt of tropical Africa, now inhabited by the gorilla and chimpanzee had extended a thousand miles farther towards Cape Colony than it now does and that the great Kalahari desert was at one time green with vegetation and covered with forest, then there would be no difficulty of explaining how anthropoid apes… came to occupy the district which is now British Bechuanaland… Here Professor Dart meets us with the objection that there is no reason to think that the forest belt ever did extend much farther south than it now does, and that in past geological periods the Kalahari was in no sense a jungle country… *Nay, it would be legitimate to cite the discovery of the Taungs skull as evidence that South Africa had at one time been covered with jungle.* We accept the fossil remains of Arctic species as evidence of a former severity of climate. On the same grounds we should accept the fossil remains of an anthropoid ape as evidence of a vegetation in South Africa which suits anthropoid needs.([47], p. 114, our italics)

Others were slightly more supportive. Richmond [48] summarized the 1926 correspondence between Dart and his former mentor Elliot Smith when the latter was busy preparing a revised version of his essay collection *The Evolution of Man* [49]. Elliot Smith assured Dart that when the revised edition was published he would ‘try to put straight before the public here your case in respect of *Australopithecus*’. In the event, although Elliot Smith praised Dart’s find as ‘exceptionally important and interesting’ [49, p. 62], he stopped short of endorsing the latter’s claim that *Au. africanus* was what we now refer to as a hominin, instead suggesting that Taung belonged to a ‘genus of Apes revealing interesting points of likeness to Man’ (*ibid.*, p. 64). Elliot Smith doubted Dart’s arguments for bipedality, but he was impressed with the human-like aspects of the facial and endocast morphology of the Taung juvenile and, like Dart, he argued that this likely revealed *Au. africanus* had been ‘emancipated from the necessity of living in forests’ (*ibid.*, p. 62).

By the 1950s, the genus *Australopithecus* had become more widely accepted as a member of the human lineage [50,51] and spurred on by the greatly expanded fossil sample from Sterkfontein and other southern African sites, researchers proposed new models for early human evolution that differed from their predecessors by being more theoretical and drawing more directly from the growing field of ecology. The new models also shifted away from environmental forcing (e.g. a directional shift from forest to savannah) as a ‘catch-all’ explanation in favour of more clearly formulated hypotheses linking what was then known of early hominin morphology to ecology (e.g. [52,53]). Detailed environmental data for the southern African hominin-bearing sites were essentially limited to the faunal assemblages at this time, although other lines of evidence began to emerge towards the end of the decade (e.g. [54] sedimentological analysis and climate reconstructions for Sterkfontein, Makapansgat and other sites).

The 1960s and 1970s saw major gains in our understanding of the environmental context of human evolution along with an expansion of the early hominin fossil record into eastern Africa, which eventually produced remains far more ancient than those recovered in the first half of the twentieth century. A key figure in these advances was Francis Clark Howell, then a young professor of anthropology at The University of Chicago. Howell’s 1959 *Science* paper ‘The Villafranchian and Human Origins’ [55] set the agenda for research on hominin palaeoecology for the next several decades. In this, Howell drew on several proxy records (e.g. marine molluscs, pollen, evidence for glaciation) to construct multi-million-year time series of environmental change that he aligned with faunal and hominin fossil and archaeological records, concluding that ‘Man’s bipedalism and the use of tools appeared during times of faunal change’ (*ibid*., p. 831). Although today we are more cautious about aligning regional and global changes in palaeoclimate with macroevolutionary ‘events’ in hominin evolution—such as the appearance and disappearance of taxa—and then suggesting that the former ‘caused’ the latter (aka ‘squiggle matching’) [56,57], Howell’s willingness to consider a role for climate was revolutionary at the time and the correlative approach remained influential for decades (e.g. [58–60]). Howell was also the primary architect behind the rise of interdisciplinary palaeoanthropology—something that arguably had been pioneered by Dorothy Garrod decades earlier [61]. Howell’s co-organization of a Wenner–Gren symposium on ‘African Ecology and Human Evolution’ in 1961, and his involvement in the edited volume that followed 2 years later [62] was crucial, as was his leading role in setting the research agenda of the International Omo Research Expedition (IORE), a multinational field campaign undertaken in the late 1960s and early 1970s that focused on the Plio-Pleistocene fossil deposits of the lower Omo Valley, Ethiopia. Howell, recognizing that advancing our understanding of human evolution required moving beyond simply studying the hominin remains and artefacts, brought together teams comprising sedimentologists, geochronologists, palaeontologists, palynologists and other specialists in the Earth sciences to determine the geological age of the IORE discoveries [63] and to reconstruct their palaeoenvironmental context. This phase in the history of human origins research laid the foundation for palaeoanthropology to develop into the highly interdisciplinary field it is today, and it is difficult to overstate Howell’s contribution to this shift in emphasis.

## 5. Early hominin evolution in Africa: Where do we stand a century after Taung?

A century after the Taung juvenile’s discovery, where does our current understanding of early hominin evolution stand? At what stage in human evolution did ‘sharpened wits’ and bipedalism appear? To answer this question, one must appreciate that molecular systematics now firmly places chimpanzees and bonobos as the closest living relatives of modern humans, with their respective clades (aka lineages) having diverged in Africa 10 to 6 Ma [64–66]. Because the current oldest fossils of *Australopithecus* date to just beyond 4 Ma, we must therefore look to a handful of late Miocene to early Pliocene genera—*Sahelanthropus, Orrorin, Ardipithecus*—that have been proposed as earlier representatives of the hominin clade. The systematic relationships of *Sahelanthropus*, *Orrorin* and *Ardipithecus* are the subjects of ongoing debate ([67–71]; in press), but these putative early hominins constitute the only relevant fossil samples from Africa that are close in time to the human–chimpanzee divergence.

The geologically oldest of these taxa, *Sahelanthropus tchadensis*, is known from 7 to 6 Ma deposits at Toros-Menalla, Chad [72]. This species comprises a relatively complete, but crushed, cranium, isolated teeth and jaws, and recently described limb bones. Hominin-like features of the cranium include a relatively short and more vertically oriented face, reduced C/p3 honing complex (i.e. the dental configuration that most non-human apes possess for sharpening the canines when the upper and lower toothrows occlude) and an anteriorly shifted foramen magnum suggesting that the head was held more upright than in chimpanzees and most bonobos. The morphology of the recently described *S. tchadensis* femur has been used by different groups to argue for [70] and against ([69,71]; in press) habitual bipedalism, whereas the forearm evidence indicates climbing [70] or knuckle-walking [73] capabilities.

In eastern Africa, *Orrorin tugenensis* is known from 6 to 5.7 Ma deposits in the Tugen Hills of Kenya [74,75]. The dental sample includes a relatively large upper canine that does not exhibit wear consistent with a C/p3 honing complex. Three partial femora come from the same fossil deposits as the *Orrorin* craniodental remains, the most complete of which has been argued to be an intermediate in morphology between Miocene apes and Plio-Pleistocene hominins [76], or similar to *Australopithecus* [77].

Finally, the genus *Ardipithecus* contains two species best known from the Awash Valley of Ethiopia. A small collection of 5.8 to 5.2 Ma fossils from the Adu-Asa Formation are attributed to *Ardipithecus kadabba*, whereas a much larger 4.5 to 4.3 Ma sample from the Sagantole Formation is attributed to *Ardipithecus ramidus*. The *Ar. kadabba* sample subsumes teeth showing a partially functional C/p3 honing complex similar to that seen in female apes, whereas some features of a pedal phalanx indicate a modern human-like toe-off mechanism during bipedal locomotion [78,79]. The younger of the two species, *Ar. ramidus*, is known from a relatively complete skeleton and more fragmentary remains of dozens of other individuals from the ~4.4 Ma site of Aramis in the Middle Awash study area. Though functional morphological interpretations are trenchantly debated (e.g. [80,81]), *Ar. ramidus* combines fore- and hind-limb adaptations for arboreality (e.g. long arms and a grasping big toe) with pelvic and hindlimb adaptations for bipedalism, and aspects of its skull and canine morphology are shared with later *Australopithecus*, albeit in a somewhat more primitive form [82–84]. Overall, the earliest putative members of the hominin lineage—*Sahelanthropus, Orrorin* and *Ardipithecus*—share a preponderance of ape-like morphology, but each taxon has been argued to possess one or more features that uniquely link them with later hominins. Whatever their taxonomic status, they suggest that bipedalism and reduced canines likely appeared early in human evolution, whereas the chimpanzee-sized brains of *S. tchadensis* and *Ar. ramidus* point to encephalization developing much later.

By 4.2 Ma, the earliest fossils of *Australopithecus* from Ethiopia [85] and Kenya [86] document a significant departure in craniodental anatomy from the earlier putative hominins. Key changes seen in early *Australopithecus* include a reduced anterior dentition with even smaller and less sexually dimorphic canines, a relatively larger crowned and more thickly enamelled postcanine dentition, and facial and mandibular features (e.g. thicker mandibular corpora) signalling a greater overall robusticity of the masticatory apparatus [87]. In terms of the postcranial skeleton, *Australopithecus* presents the first unanimously accepted evidence for hominin bipedalism [88,89]. Understanding which, if any, of the putative hominin taxa from the late Miocene to early Pliocene might have been ancestral to *Australopithecus* remains an ongoing challenge [15].

The reconstruction of the palaeoenvironmental context of early human evolution continues to be a contentious topic. The only general points of agreement are that ‘open veldt country’ in the sense of Dart [11]—open grassland—is almost certainly not the environment within which hominins emerged, and that broad characterizations of past environments as either ‘savannah’ or ‘forest’ are unhelpful over-simplifications. Nearly all present-day environments in sub-Saharan Africa are mosaics of vegetation types that spatially and temporally vary with the effects of climate, fire, herbivory and local geology [90,91]. This was undoubtedly also true of the past, with the added complications that our palaeo-proxies record information about ancient environments at different spatial and temporal scales [56,92,93] and that environments of the past may not necessarily have present-day analogues [94,95]. It is therefore unsurprising that, for example, the environments of *S. tchadensis* at Toros-Menalla have been characterized by phytolith analysis as a forest–palm grove mosaic with woodlands [96], while the faunal evidence [97,98] points to a more grass-dominated landscape. The earliest putative hominins and *Australopithecus anamensis* are both characterized by isotopically ape-like diets (C_3_ plants, presumably fruit and leaves) [99] and forelimb morphology suggesting significant arboreality [82], so more recent models locate hominin origins in the context of relatively wooded environments (e.g. 82,83,100]). Observations of how great apes move in their natural habitat have prompted the suggestion that bipedalism might have arisen as an above-branch arboreal adaptation that was only later co-opted for terrestrial locomotion [101–103]. While the studies cited above provide relevant referential data, the existence of non-analogue anatomical packages [82,88] and ecosystems [94] in the past cautions against extrapolating the behaviours of extant great apes to early hominin evolutionary history.

## 6. Summary

In 1863, in the course of reviewing the available fossil evidence for human origins, and impressed with the rather modern aspect of the known fossil sample (essentially the Feldhofer Neanderthal and Upper Palaeolithic *Homo sapiens*), Thomas Henry Huxley asked: ‘Where, then, must we look for primæval man?… In still older strata do the fossilized bones of an Ape more anthropoid, or a Man more pithecoid, than any yet known await the researches of some unborn palaeontologist?’ [1, p. 184]. There is now universal agreement that Africa hosted the earliest stages of human evolution, with Raymond Dart, the Taung juvenile and the recognition of *Au*. *africanus* providing the first legitimate scientific response to Huxley’s questions. The past 100 years have witnessed substantial gains in our understanding of early human evolution in Africa, including the sequence and approximate chronology of key events (e.g. bipedalism and small canines appear long before stone tool use and encephalization) and validation that *Australopithecus* is the probable ancestral group from which the genus *Homo*—and ultimately modern humans—derived.

Much about the human evolutionary story has changed over the past century, but one reality has not. Despite crucial developments in molecular and primatological studies, fossils and their context remain the primary evidence for understanding our origins.

## Data Availability

This article has no additional data.

## Footnotes

^1^
The name of the small town, and thus the fossil’s discovery site, was originally Taungs. It was later changed to Taung, but it is not clear when this change occurred.
